# Identification of Small RNAs Associated with Salt Stress in *Chrysanthemums* through High-Throughput Sequencing and Bioinformatics Analysis

**DOI:** 10.3390/genes14030561

**Published:** 2023-02-23

**Authors:** Jiefei Nai, Tieming Ma, Yingjie Liu, Yunwei Zhou

**Affiliations:** 1 College of Forestry and Grassland Science, Jilin Agricultural University, No. 2888, Xincheng Street, Changchun 130118, China; 2 College of Landscape Architecture, Northeast Forestry University, No. 26, Hexing Road, Harbin 150006, China

**Keywords:** *Chrysanthemum*, salt stress, miRNA, high-throughput sequencing

## Abstract

The *Chrysanthemum* variety “Niu 9717” exhibits excellent characteristics as an ornamental plant and has good salt resistance. In this study, this plant was treated with 200 mM NaCl for 12 h followed by high-throughput sequencing of miRNA and degradome. Subsequently, the regulatory patterns of potential miRNAs and their target genes were searched to elucidate how *Chrysanthemum* miRNAs respond to salt. From the root and leaf samples, we identified a total of 201 known miRNAs belonging to 40 families; furthermore, we identified 79 new miRNAs, of which 18 were significantly differentially expressed (*p* < 0.05). The expressed miRNAs, which targeted a total of 144 mRNAs in the leaf and 215 mRNAs in the root, formed 144 and 226 miRNA–target pairs in roots and leaves, respectively. Combined with the miRNA expression profile, degradome and transcriptome data were then analyzed to understand the possible effects of the miRNA target genes and their pathways on salt stress. The identified genes were mostly located in pathways related to hormone signaling during plant growth and development. Overall, these findings suggest that conserved and novel miRNAs may improve salt tolerance through the regulation of hormone signal synthesis or expression of genes involved in hormone synthesis.

## 1. Introduction

*Chrysanthemum* is a perennial root herb of the *Asteraceae* family, a traditional Chinese flower, and one of the four major cut flowers worldwide. It has good economic and medicinal values. It also plays an important role in urban greening and landscaping [1]. Secondary salinization of global soils is aggravated, resulting in salt stress that affects the growth and development of plants and leads to plant death. Already 100 million hectares of saline–alkali land is present in China; therefore, how to use these large areas of saline–alkali land to achieve sustainable development in the agricultural industry and improve the ecological environment by improving the salt tolerance of plants has become an urgent issue to be resolved [2,3].

The materials (*Chrysanthemum* (*Dendranthema* × *grandiflora*) (2n = 6x = 54)) used in this study were the hybrid of Beijing *Chrysanthemum* and ground *Chrysanthemum*, subsequently the hybrid was obtained by spaceflight mutagenesis after hybridization [4]. Previous studies have shown that plants initiate their salt tolerance mechanisms to adapt to and resist salt stress, such as reactive oxygen species removal [5], related hormone accumulation [6], activation of signaling pathways associated with salt tolerance [7], and miRNA regulation [8]. Therefore, more salt-tolerant *Chrysanthemum* varieties can be reasonably used to study salt tolerance mechanisms.

MiRNAs are a class of endogenous small-molecule RNAs targeting the mRNAs that control degradation or inhibit translation [9,10] and miRNAs usually play a role in negatively regulating the gene expression at the post-transcriptional level, controlling the expression of many genes involved in various biological and metabolic pathways, and promoting plant growth and development [11,12,13]. In particular, miRNA plays an important role in dealing with abiotic stresses, such as salt stress [14,15], heavy metal stress [16], and high-temperature stress [17,18]. Sequencing techniques for small RNAs primarily aimed at detecting miRNAs are now commonly used in plant epigenetics. The discovery of miRNAs provides researchers with new perspectives on plant salt tolerance mechanisms.

Salt stress is a type of abiotic stress. When plants are subjected to salt stress, they initiate a regulatory process involving miRNAs to cope with the stressful environment. In general, salt stress first acts on miRNAs and then on the corresponding target genes, and the interaction of these miRNAs with target genes enables life processes, including seed embryonic development, lateral root and main root growth, flower organ formation, and various hormone syntheses associated with growth and development, to adapt to the stress environment. For example, Arabidopsis miR156 directly targets AtSPL10 and AtSPL11 [19,20]. Under salt stress conditions, miR156 expression increased and improved the seed germination rate by regulating early embryonic development in plants, thereby improving salt tolerance and germination rate of alfalfa seeds overexpressing miR156 [21]. Salt resistance in rice and *Arabidopsis thaliana* overexpressing miR393 was improved via TIR1 (*Transport inhibitor response protein 1*) as the target gene of miR393, and miR393 and the target gene regulated the ability of seeds to germinate by participating in the expression of salt tolerance-related genes, *P5CDH* and *SR05* [15]. *m*iR159 expression in maize under salt stress conditions was upregulated and affected ABA(Abscisic acid) signal regulation through joint action with its target gene MYB(v-myb avian myeloblastosis viral oncogene homolog), thereby increasing the seed germination rate and enhancing the salt resistance of maize [22,23]. Salt stress can induce upregulated expression of miR169 in rice, which selectively degrades the target gene *NF-YA* (nuclear factor-YA), thereby reducing its sensitivity to ABA and overexpression of miRNA169 in *Arabidopsis* increased its salt tolerance [8]. The overexpression of rice *Os-miR319* in bentgrass was found to improve salt resistance in the transgenic material, increase the waxy layer content and water holding capacity of the leaves, and reduce Na^+^ absorption. Moreover, miR319 regulated the morphological formation and growth and development of plant leaves by targeting some members of the TCP (Teosinte Branched1/Cycloidea/Proliferating Cell Factors) transcription factor family [8,19,24].

In addition, miRNAs can improve the salt resistance of plants by regulating reactive oxygen species elimination processes in cotton [25], Phaseolus vulgaris [26], *Arabidopsis*, European aspen [27], and other plants exhibiting enhanced structural protection activity, such as Arabidopsis miR398 targeting *CSD1* and plastoid *CSD2* of the cytoplasm [28], small bowl moss miR1073 targeting Cu-Zn-CSD [29], and cowpea miR408 targeting peptide chain release factor [30]. These miRNAs have been shown to enhance the protective effects on the cell membrane by regulating reactive oxygen species processes, thereby improving the salt resistance of plants.

Finally, miRNAs can also improve salt resistance by participating in protein hydrolysis, facilitating cellular life activities, and regulating signaling pathways, such as the salt-grown plant salt spike wood, miR894, and its target gene *UBE2H* to participate in proteolysis under stress; miR2867 participating in the DNA repair process under stress through the action of the target gene *RFC*;miR5077 and miR2619 participating in the Ca^2+^ signaling pathway through the action of their target genes *PLC* and *PPP3C*. The signaling pathway regulation process of MAPK (mitogen-activated protein kinase), miR159, and its target gene *ATM (ataxia telangiectasia-mutated)* is involved in apoptosis regulation under salt stress conditions [31,32].

In recent years, a study on the molecular mechanisms of miRNAs has provided a new understanding of plants’ salt tolerance mechanisms; however, miRNAs of different plant materials still greatly differ in function and salt resistance mechanisms. Therefore, exploring the salt tolerance mechanism from the miRNA perspective might improve the salt tolerance ability of *Chrysanthemums* and provide a basis for the effective utilization of *Chrysanthemum* gene resources and breeding of resistant varieties.

## 2. Materials and Methods

### 2.1. Plant Materials and Salt Treatment

*Chrysanthemum × grandiflora “NIU9717”* was used in this study. All seedlings were produced via tissue culture and planted in a greenhouse at Northeast Forest University (Harbin, China) under an average temperature of 26 °C, a light/dark cycle of 16/8 h, and relative humidity of 65%. When plants reached 7–8 cotyledons, they were randomly classified into four groups, each comprising three replicates. When the plants reached 9–10 cotyledons, salt treatment was initiated by adding 60 mL of 200 mM/L at 8 am the same day. Solution without any added NaCl was used as the control. After 12 h of salt treatment, roots and shoots of an individual plant from each treatment and control group were harvested as one replicate. We obtained four types of samples: roots under control conditions (SCK-R), leaves under control conditions (SCK-L), roots under 200 mmol/L salt stress (S200-R), and leaves under 200 mmol/L salt stress (S200-L). Twelve samples (three replicates per sample type) were frozen in liquid nitrogen at −80 °C until RNA extraction.

### 2.2. Small RNA Sequencing and miRNA Identification

Approximately 1 µg of total RNA was used to generate small RNA libraries in accordance with the TruSeq Small RNA Sample Prep Kit protocol (Illumina, San Diego, CA, USA). We then processed single-end sequencing data (36 bp and 50 bp) on an Illumina Hiseq2500 platform (LC-Bio, Beijing China). Data were analyzed following the procedures recommended by LC Sciences Service, with modification to predict plant hairpin structures. To remove adapter dimers, junk, low complexity, common RNA families (rRNA, tRNA, snRNA, snoRNA), and repeats, the raw reads were subjected to ACGT101-miR v3. 5 (LC Science, Houston, TX, USA). To identify known miRNAs and novel 3p- and 5p-derived miRNAs, the remaining clean reads with a length of 18–25 nucleotides were mapped to specific species precursors in MiRBase 22.0 using BLAST. Novel miRNA candidates were defined as unique sequences that mapped to the other arm of known specific species precursor hairpins but opposite the annotated mature miRNA-containing arm. The remaining sequences were then mapped to the other selected species precursors in MiRBase 22.0 using BLAST. The mapped pre-miRNAs were analyzed against the specific species genomes using BLAST to determine their genomic locations. Length variation at the 3′ and 5′ ends and one internal mismatch were allowed in the alignment. We defined the above two types as known miRNAs. The unmapped sequences were analyzed against the specific genomes via BLAST. Using RNAfold (http://rna.tbi.univie.ac.at/, accessed on 1 May 2021), hairpin RNA structures were predicted from the flank 120 nt sequences. The expression of miRNAs was then normalized using a common set of sequences among all samples [33]. Based on the normalized deep-sequencing counts, differential expression of miRNAs was analyzed using Fisher’s exact test or Chi-squared test with 2 × 2 or n × n contingency tables, Student’s t-test, or analysis of variance, as appropriate. The threshold for statistical significance was set at <0.05.

### 2.3. Degradome Sequencing and Target Identification

Equal amounts of the 12 frozen samples were pooled together for RNA extraction. A degradome library was prepared from approximately 20 µg of the pooled total RNA sample. Total RNA was extracted and captured on beads and connected with a 3′–5′ adaptor. The whole library was constructed using mixed reverse transcription of biotinylated random primers and mRNA and amplified via PCR. Subsequently, the constructed library was sequenced using an Illumina HiSeq 2500 (LC-Bio, Beijing, China). Raw data obtained via sequencing were used to predict miRNA target genes using the CleaveLand 3.0 [34] and ACGT301-DGEv1.0 programs (LC Sciences, Houston, TX, USA). Given the abundance of the resulting mRNA tags relative to the overall profile of the degradome reads that matched the target [35], the target genes were divided into five categories, namely, 0, 1, 2, 3, and 4.

Enrichment analysis of the candidate target genes was performed using Gene Ontology (GO) functional terms and the Kyoto Encyclopedia of Genes and Genomes (KEGG). A two-tailed Fisher’s exact test was applied to identify the enriched terms with corrected *p*-values of < 0.05, considered statistically significant. The background consisted of all miRNAs in the respective databases. Functional annotation was performed using sequence similarity.

### 2.4. The Verification of Authenticity of High-throughput Sequencing Data via qRT–PCR

To validate the reliability of the high-throughput sequencing data, qRT–PCR was performed for 10 selected miRNAs. Reverse transcription and qRT–PCR were performed using a Mir-X miRNA First-Strand Synthesis Kit (TaKaRa, Tokyo, Japan) and 2× TSINGKE Master qPCRMix (SYBR Green I, Qingke Biotech, Beijing, China) according to the manufacturers’ instructions, respectively. U6 sRNA was used as the reference gene for normalizing miRNA expression. The sequences of the primers used for qRT–PCR in this study are presented in Table S8. The relative expression levels of miRNA were calculated using the 2^−ΔΔCt^ method [36]. Three technical replicates were performed for each reaction. Correlation analysis of miRNA expression profiles between high-throughput sequencing and qRT–PCR data was performed using R version 3.1.1.

## 3. Results

### 3.1. MiRNA Sequence Analysis

For the SCK-L and SCK-R samples of the two control groups and the S200-L and S200-R samples of the experimental control group, 12 small RNAs (miRNAs) including 3 biological replicates were constructed. A total of 38,138,140, 42,938,820, 37,046,602, and 48,942,450 raw reads for the SCK-L, SCK-R, S200-L, and S200-R samples were obtained, respectively. After using high-throughput sequencing and removing adapter sequences, poly-A sequence, low-mass data, and fragments of <18 nt and >25 nt, 30,744,934, 25,433,950, 31,638,068, and 33,960,350 clean reads were obtained from SCK-L, SCK-R, S200-L, and S200-R. The clean unique reads were compared to the Rfam database and annotated as tRNA, rRNA, snRNA, scRNA, snoRNA, and repeat sequences to obtain corresponding miRNAs (Table 1). The sRNA length in this data was 21–24 nt, with 21 nt and 24 nt being the most abundant, and no significant difference in sRNA length was found among the 12 libraries (Figure S1). These results are consistent with those of planting sRNAs obtained in other studies. The length distribution of unique miRNA is concentrated at 21 nt (53.11%). When comparing the relevant noncoding RNA from the 3′ to the 5′ end stored in the mibase, Rfam, and Rephase databases to search for and delete rRNA, tRNA, SnRNA, and snoRNA sequences as well as predict the novel miRNA, we detected the remaining undescribed miRNAs that did not match the transcriptome of any library and made secondary structure predictions. All sRNA sites that can be folded into secondary structures are considered candidate sites for potential new miRNAs. Through this method, a total of 280 miRNAs were identified, 201 of which were known miRNAs belonging to 40 families corresponding to 193 pre-miRNAs and 79 new miRNAs corresponding to 67 pre-miRNAs.

### 3.2. Identification of Conserved miRNAs

**Figure 1:**
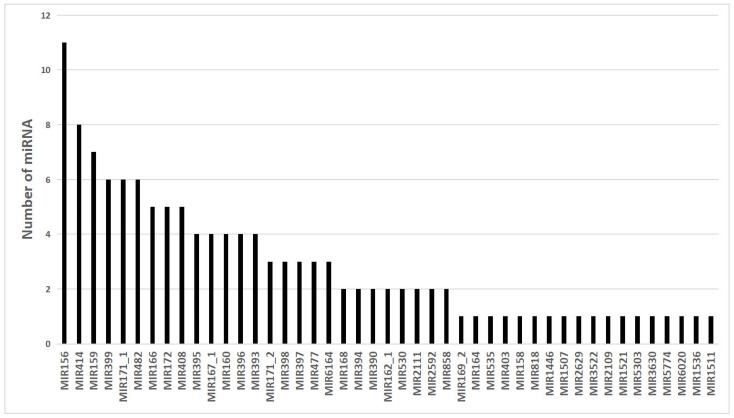
Distribution of conserved miRNAs in the miRNA family.

**Figure 2:**
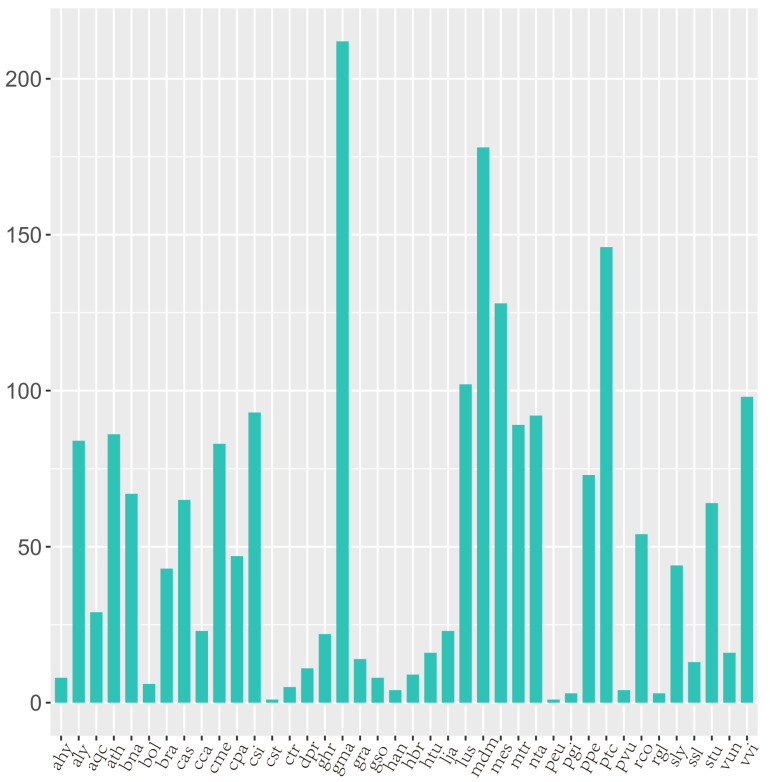
Similarities between known miRNAs in *Chrysanthemum* and other plants.

To identify conserved miRNAs, unique sRNA sequences generated from all libraries were aligned to known plant miRNAs on miRbse20.0, allowing up to two base mismatches. Among four sets of sRNAs (SCK-L, S200-L, SCK-R, and S200-R), a total of 201 miRNAs were identified from 40 highly matched families. Most conserved miRNAs were 21 nt in length, and precursors of conserved miRNAs were 20–169 nt in length. These miRNAs were widely expressed from <10 reads to >100,000 reads (Table S1). For example, miR166, miR159, and miR396 were abundantly expressed in *Chrysanthemum* leaves and roots of both the treated and control groups. Some miRNA families identified in Arabidopsis, soybean, and other plants, such as miR156, miR171, and miR396, were highly conserved. However, the number of members in these conservative miRNA families differs, e.g., miR156, miR159, miR399, miR171-1, miR414, and miR482 families have more than five members, but most families have only one (Figure 1). Meanwhile, miRNAs of Radix officinalis had significant similarity with several known miRNAs in other plants. For example, miRNAs showed significant similarity with 212 known miRNAs in soybean (*Glycine max)*, 178 miRNAs in apple (*Malus domestica*), and 146 in poplar (*Populus trichocarpa*). The known miRNAs showed significant similarity (Figure 2).

### 3.3. Identification of Novel miRNAs

**Figure 3:**
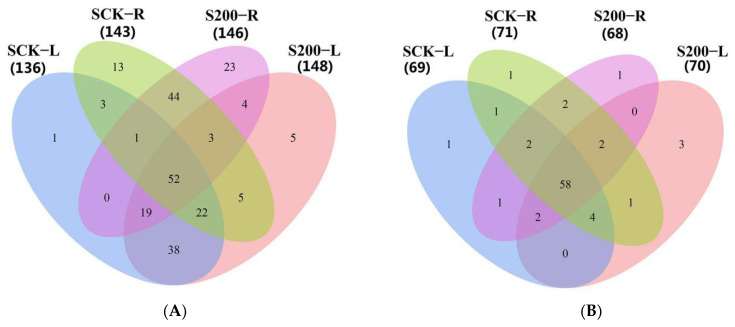
Expression values of conserved miRNAs and novel miRNAs in the roots and leaves of the four libraries. (**A**) The number of conserved miRNAs:SCK-L vs. S200-L;SCK-R vs. S200-R. (**B**) The number of novel miRNAs:SCK-L vs. S200-L;SCK-R vs. S200-R.

A total of 79 novel miRNAs were identified in four sets of libraries. The length of the novel miRNAs was between 20 and 24 nt, with 24 nt being the most abundant. Precursors of these novel miRNAs were between 77 and 258 nt in length. Using Mfold calculations, the minimum free energy and its indices of the predicted pre-miRNAs ranged from −26 to −127.20 kcal/mol and 0.9 to 1.90 kcal/mol, respectively (Table S2). The predicted stem-loop structure of some novel miRNAs, as shown in Figure S2(1) and Figure S2(2), can form a stable stem-loop structure and meet conditions for miRNA formation. These findings were consistent with observations for other plant precursors of miRNAs. Analysis of nucleotide position specificity revealed a clear preference for uracil (U) in the first position, whereas guanine (G) was the least abundant in 18–24 nt. Furthermore, analysis of the nucleotide preference of 21–24 nt miRNAs showed that 19–22 nt miRNAs still started with U as the base (Figure S3). This is consistent with the bias characteristic of the first base pair U of miRNA. In the four sets of libraries, 136, 148, 143, and 146 conserved miRNAs were expressed in SCK-L, S200-L, SCK-R, and S200-R, respectively. Moreover, 69, 70, 71, and 68 new miRNAs were expressed in SCK-L, S200-L, SCK-R, and S200-R, respectively. Figure 3a and Figure 3b show that the expression of most conserved miRNAs is relatively stable in different tissues of *C. Niu9717*, and plants can conserve miRNA transcription under salt stress, which is not obvious in novel miRNAs.

### 3.4. Identification of Differentially Expressed miRNAs

Differential expression levels of miRNAs in roots and leaves under salt stress were identified by reading counts and it was found that 220 and 227 miRNAs were differentially expressed in the leaves and roots, respectively. In the S200-L vs. SCK-L group, 75 novel miRNAs and 145 conserved miRNAs were differentially expressed in the leaves, of which 120 were upregulated and 100 were downregulated. Seven miRNAs exhibited significant differential expression (three upregulated and four downregulated) (Table 2). In the S200-R vs. SCK-R group, 75 novel miRNAs and 152 conserved miRNAs were differentially expressed in the roots. Among them, 124 were upregulated, 103 were downregulated, and 12 were significantly differentially expressed (4 novel miRNAs and 8 conserved miRNAs) (6 upregulated and 6 downregulated) (Table 3).

### 3.5. Validation of miRNA Expression via qRT–PCR

**Figure 4:**
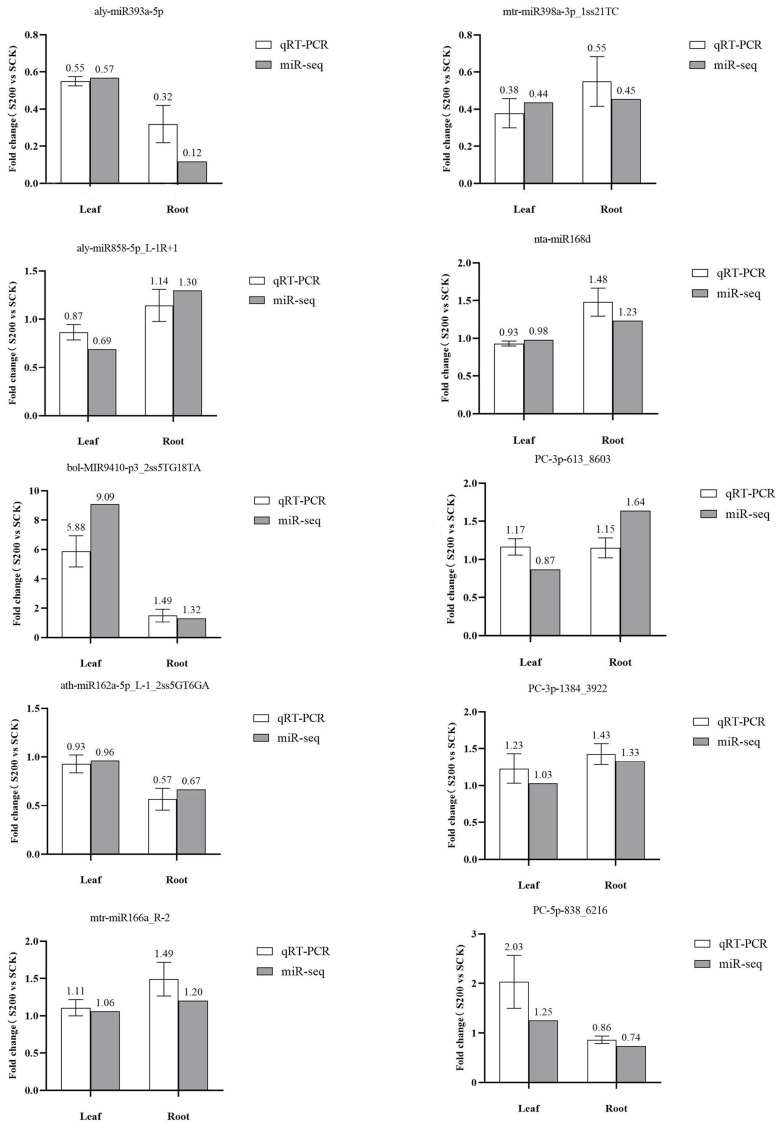
qRT–PCR analysis results of ten miRNAs with significant differences in the roots and leaves of *Chrysanthemum* under salt stress.

To verify the reliability of miRNA sequencing results and explore the expressive characteristics of miRNAs associated with salt tolerance, ten miRNAs with significant differences of expression levels in the roots and leaves were selected and analyzed using qRT–PCR. The expression levels of the following ten miRNAs under CK and salt stress conditions were determined: aly-miR393a-5p, aly-miR858-5p_L-1R+1, ath-miR162a-5p_L-1_2ss5GT6GA, bol-MIR9410-p3_2ss5TG18TA, mtr-miR166a_R-2, mtr-miR398a-3p_1ss21TC, nta-miR168d, PC-3p-613_8603, PC-3p-1384_3922, and PC-5p-838_6216. The results showed that except for PC-3p-613_8603, the expression patterns of the remaining nine miRNAs selected in qRT–PCR experiments were consistent with those detected via high-throughput sequencing (Figure 4). Most high-throughput sequencing results can be verified via qRT–PCR, indicating that the sequencing data were authentic and reliable.

### 3.6. Degradome Sequencing and GO Enrichment Analysis

A total of 20,974,590 (DCK-L), 20,245,902 (DCK-R), 20,417,390 (D200-L), and 31,251,146 (D200-R) raw reads in the four degradome libraries, with 2,796,648 (48.84%), 3,625,524 (43.38%), 2,562,083 (46.89%), and 4,215,725 (39.03%) unique mapped reads (Table S3), respectively, and 45,632 transcripts were used to detect miRNA cleavage sites. A total of 232 miRNAs (161 conserved miRNAs and 75 novel miRNAs) targeted 4091 transcripts in the leaves, and 250 miRNAs (175 conserved miRNAs and 75 novel miR-NAs) targeted 5753 transcripts in the roots (Table S4). Among them, only ten targetable transcripts (PC-3p-243205_24, PC-3p-45948_186, PC-3p-4615_1376, PC-5p-759420_5, ath-miR162a-5p_L-1_2ss5GT6GA, cca-miR156c_R+1, mtr-miR156e, ppe-miR399b_L-2R+2, ppe-miR535a, and stu-miR156a_R+1) were detected in the roots and only one targetable transcript (peu-MIR2916-p5_2ss4AG19TG) was detected in the leaves. gma-MIR169g-p3_2ss10TG20TG was the single miRNA that degraded the highest number of transcripts, with 521 and 698 transcripts detected in the leaves and roots, respectively. One target gene is regulated by multiple miRNAs, for example, TRINITY_DN121394_c1_g3 is simultaneously targeted by four different miRNAs (PC-3p-268483_20, PC-3p-427146_10, ath-MIR414-p3_2ss14AC17AC, and mdm-MIR169k-p3_2ss15CG17TA). Among these identified targets, 142, 23, 908, 582, and 731 belonged to the categories 0, 1, 2, 3, and 4 in the leaves, respectively, and 181, 22, 1639, 1129, and 1209 belonged to the categories 0, 1, 2, 3, and 4 in the roots, respectively (Table S5). Furthermore, according to sequencing results of the degradome, the same target gene corresponding to miRNA may have different classifications in the roots and leaves under salt stress. For example, one target gene of aly-miR393a-3p, TRINITY_DN123013_c2_g2, has a rank of 2 in the leaf and 3 in the roots.

**Figure 5:**
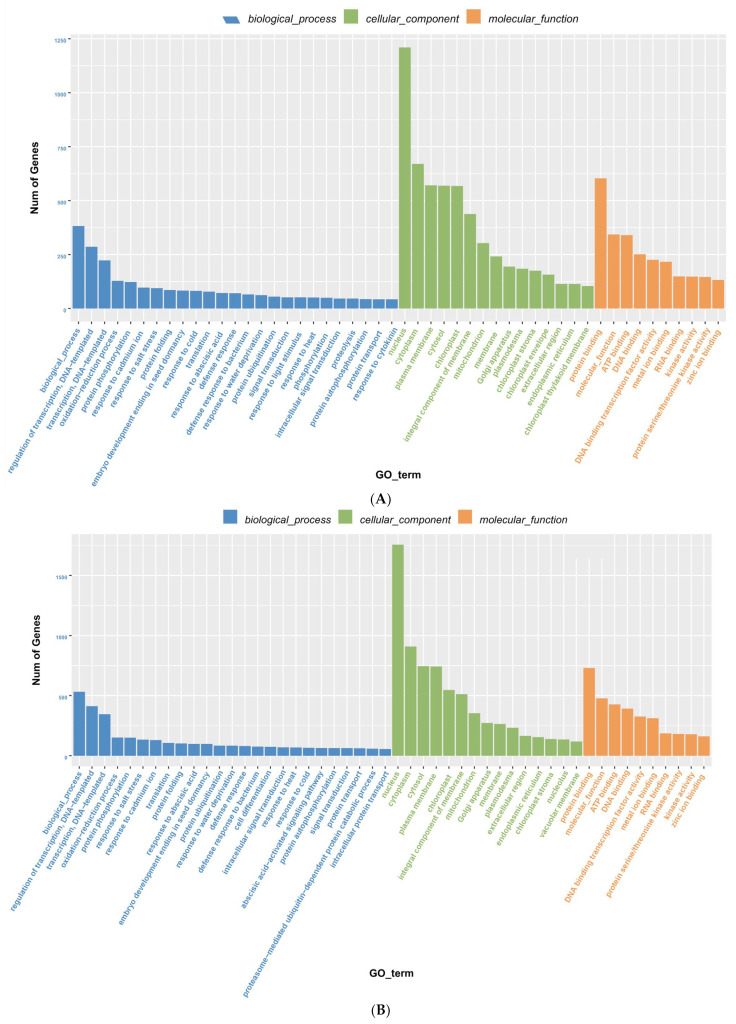
GO functional annotation of target genes between (**A**) leaves and (**B**) roots in *Chrysanthemum.* The x-axis represents the GO functional annotation. The y-axis represents the total number of targets annotated.

Based on GO functional annotations, target genes were classified into three categories: biological processes, molecular functions, and cellular components. Genes targeted by differentially expressed miRNAs in the S200-L vs. SCK200-L and S200-R vs. SCK200-R libraries were the most annotated biological processes, followed by molecular functions and cellular components (Table S6). Moreover, during the cellular processes, the number of genes for protein binding, molecular function, and ATP binding rank among the top three; among the molecular functions, the number of genes expressed in the nuclear processes are the most common (>1000 unigenes); during the biological processes, transcriptional and DNA template regulatory annotations are for more genes (Figure 5). Among the GO terms of TOP20, the root and leaf nuclei, cytosol and protein binding differences were the most significant (Figure S4). These data indicate that the target genes corresponding to miRNAs respond to stress by changing the cell morphology and protein binding.

### 3.7. Analysis of the KEGG Pathway

**Figure 6:**
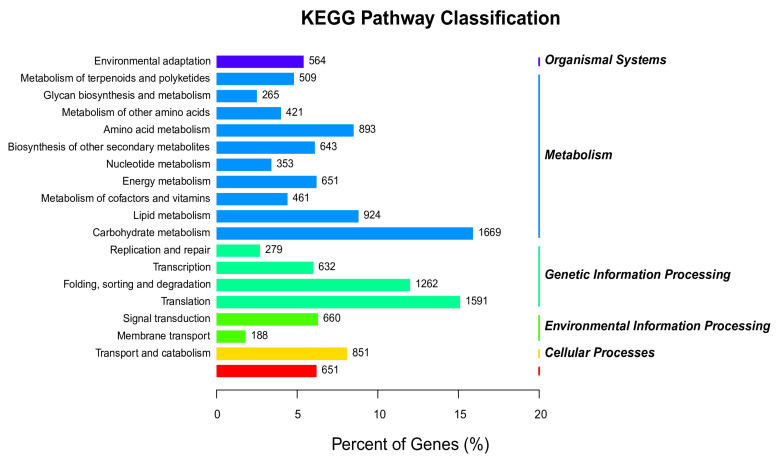
Enrichment analysis of the KEGG pathway between leaves and roots in *Chrysanthemum* under salt stress. The x-axis represents the percentage of targets annotated to the pathway out of the total number of targets annotated. The y-axis represents the KEGG metabolic pathway.

Based on the KEGG database, the differentially expressed miRNAs in leaves were involved in 132 metabolic networks, whereas miRNAs in roots were involved in 133 metabolic networks. Four pathways were separately expressed in roots and leaves (ko00471, ko00966, ko00430, ko00254, and ko0096 were expressed separately in the root; ko00232 ko00402, ko00523, and ko03450 were expressed separately in the leaf). These networks indicate that both root and leaf miRNAs target genes that are mainly involved in translation and carbohydrate and hydration metabolic processes in response to salt stress (Figure 6).

### 3.8. Combined Analysis of Significant miRNA-Targets

To identify the expression mode at the transcriptional level, we combined miRNA, transcriptome, and degradome data for analysis. In salt-stressed ground-grown *Chrysanthemum*, 7 miRNAs showed significant differential expression in the leaves and were predicted to target 144 different mRNAs, forming 144 miRNA–target pairs; moreover, 12 miRNAs showed significant differential expression in the roots. miRNAs with significant differential expression were predicted to target 215 different mRNAs, forming 216 miRNA–target pairs (Table S7).

**Figure 7:**
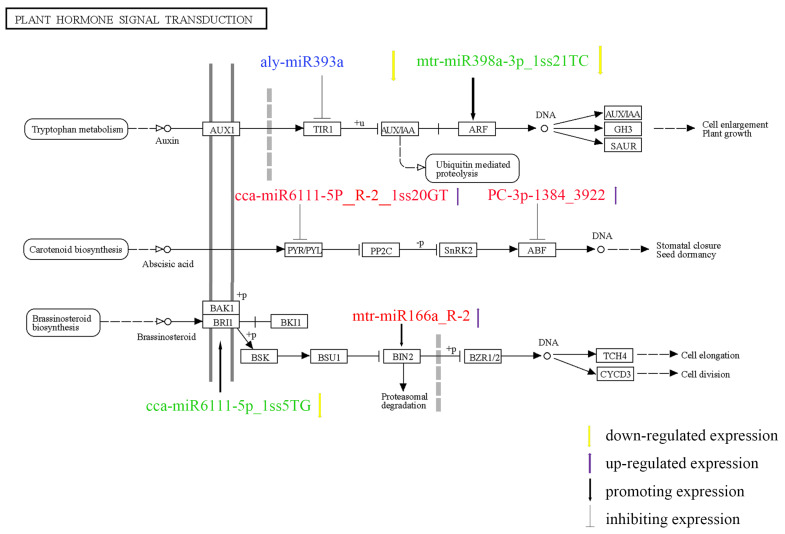
The potential regulatory system of miRNAs in ground-grown *Chrysanthemum* in response to salt stress. Green font represents significantly differentially expressed miRNAs in leaves, red font represents significantly differentially expressed miRNAs in roots, blue font represents significantly differentially expressed miRNAs in both roots and leaves.

The target genes of miRNAs with significant differential expression include transcription factors, hormone-responsive genes, DNA/RNA binding proteins, protein-coding genes, and enzymes. Data analysis and classification showed that miRNA and its corresponding target genes were mostly involved in plant hormone signal transduction, followed by RNA transport; plant–pathogen interactions; glycine, serine and threonine metabolism; ubiquitin-mediated proteolysis, mRNA surveillance pathway, and endocytosis are shown in Table 4. In general, salt stress response systems and membrane receptors sense extracellular stress signals, conduct signal transduction, induce salt-related gene expression through transcriptional regulation, and finally cause physiological changes in response to stress. Among them, salt stress signal transduction mainly includes the ABA, protein kinase, and SOS pathways. Figure 7 shows the potential regulatory system of miRNAs in ground-grown *Chrysanthemum* in response to salt stress. Most of the miRNA target genes that were differentially expressed in *Chrysanthemum* under salt stress were involved in hormonal pathways.

## 4. Discussion

### 4.1. Prediction of Novel miRNAs in Ground-Grown Chrysanthemum Using High-Throughput Sequencing

Using high-throughput sequencing technology and biological analytical methods, a total of 201 known miRNAs belonging to 40 families were obtained from the leaves and roots and 79 new miRNAs were predicted. Previous studies have found that the length of sRNA varies among different species. The sRNA length of blueberry [37], sweet potato [38], and tea tree [39] is at most 24 nt, whereas that of poplar [40], soybeans [41], and tomato [42] is at most 21 nt, and high-throughput sequencing data showed that the sRNA length in 12 databases is at most 24 nt, followed by 21 nt (Figure 1), which is similar to that of Ramat Ju [43]. The reported typical sRNA distribution patterns are extremely similar. SRNAs with different lengths are related to different functions. SRNAs with a length of 21–22 nt are mainly associated with mRNA cleavage and post-transcriptional gene silencing [44], and sRNAs with a length of 24 nt are mainly related to RNA-guided DNA methylation [45]. Studies have shown that when the MEFI is >0.85, the sequence is considered most likely to be a miRNA [46], and in this study, the minimum folding free energy index of the new pre-miRNAs was approximately between 0.90 and 1.90. It is 1.32, i.e., higher than other sRNA types [47]; thus, it can form stable miRNA.

### 4.2. Differential miRNA Profiling in Response to Salt Stress of the Ground-Grown Chrysanthemum

Under salt stress, conserved miRNAs, such as *miR393*, *miR166*, *miR398*, and *miR168*, were significantly differentially expressed. These miRNAs have been identified in plants under salt stress conditions [48]. Most miRNAs associated with salt stress response in plants are conserved; however, some miRNAs have different regulatory patterns among different species. For example, *miR393* was significantly upregulated in *Arabidopsis* [8] and rice [49] under salt stress treatment, whereas tobacco *miR393* was significantly downregulated at low concentrations [50]. In the present study, *miR393* was significantly downregulated in both leaves and roots. Furthermore, in different plant species, the expression pattern of the same miRNA will be different. For example, in this study, expression patterns of the *miR6111* family at the 3′ and 5′ ends of the *miR6111* family in the same tissue site after salt stress treatment were opposite. Expression patterns at the same end of the family are also reversed. For example, cca-MIR6111-p3 at the 3′ end was significantly downregulated, and cca-miR6111-5p_R-2_1ss20GT at the 5′ end was significantly upregulated in roots, cca-MIR6111-p3_2ss17GA19CT at the 3′ end was significantly upregulated in the leaves, and cca-miR6111-5p_1ss5TG was significantly upregulated. This is consistent with the results of a previous report that the *miR530* family in sweet potatoes showed opposite expression patterns in the roots and leaves under salt stress [38], indicating that they may have different salt stress response mechanisms and members of the same family A more complex regulatory network may be formed internally; however, the exact reason remains unclear. In addition to these conserved miRNAs, the expression of three novel miRNAs (PC-3p-153871_47, PC-3p-613_8603, and PC-3p-1384_3922) in the roots under salt stress was significantly upregulated, whereas PC-5p-838_6216 was significantly downregulated. These specifically expressed, conserved, and novel miRNAs may be involved in the regulatory network in response to salt stress of *Spathiphyllum* and regulate the gene expression and metabolic processes during salt stress.

### 4.3. Combining sRNA and Degradome Sequencing to Analyze the Role of Ground-Grown Chrysanthemum miRNAs in Response to Salt Stress

Most target genes targeting the ground-grown *Chrysanthemum* under salt stress were related to the known salt tolerance, such as mtr-miR166a_R-2, which was significantly upregulated in the roots, targeting REV and ATHB8, which are HD-ZIP III of the TF family. Studies have shown that plants overexpressing the HD-ZIPIII gene in *Arabidopsis* have stronger salt tolerance than the wild type, and this increase in salt tolerance may be related to reduced miR165/166 expression in transgenic *Arabidopsis*. Related studies [51], Josietal [48], and Yan [52] believed that upregulation of the expression of miR166 directly promotes the *ABI4* and *BG1* accumulation, thereby regulating ABA and abiotic stress response processes and controlling ABA homeostasis to improve salt tolerance. Under salt stress, the interaction relationship between mtr-miR166a_R-2, REV and ATHB8 related to ground-grown *Chrysanthemum* and whether it is directly transmitted through the ABA signaling pathway in response to salt stress needs further study.

The mtr-miR398a-5p_2ss12GC21AT and mtr-miR398a-3p_1ss21TC target genes were significantly downregulated in salt-stressed ground-grown Chrysanthemum roots and were CSD2 and CCS. Studies have shown that the main miR398 target genes in plants are superoxide dismutases (CSD) and CCS. The CSD gene is the main superoxide dismutase for plants to resist reactive oxygen poisoning [53], and CCS can transfer copper ions to CSD to balance substances in plants [54], both improve the salt tolerance of plants by improving resistance to toxic effects of reactive oxygen species in plants. For example, transgenic rice overexpressing the miR398-resistant form of Os-CSD2 showed stronger tolerance to high salt stress than *nontransgenic rice* [55]. However, miR398 expression was downregulated at 12 h in *Chrysanthemums chinensis*, whereas miR398 in *Arabidopsis* under salt stress was significantly downregulated in CSD1 after 12 h of treatment, and CSD2 was changed only after 48 h [56]. MiR398 in mustards was negatively regulated by CSD1 and CSD2. The difference in this result may be due to differential regulation among different plant species.

The significantly upregulated nta-miR168d in the roots of ground-grown *Chrysanthemum* under salt stress treatment targets the *AGO1* protein involved in miRNA biosynthesis. In *Arabidopsis*, AGO1 is involved in the miRNA-mediated mRNA cleavage process [57]. The increased miR168 expression inhibits the synthesis of target gene *AGO* protein, attenuates miRNA-mediated mRNA cleavage, and results in increased protein levels at the translational level, thereby activating and enhancing various physiological function pathways in plants [58].

Furthermore, the target gene of *miR393*, which was significantly downregulated in both roots and leaves of ground-grown *Chrysanthemum*, was the auxin receptor TIR1. Studies have shown that miR393 regulates the expression of growth hormones during seed germination by participating in the expression of salt tolerance genes P5CDH and SR05 through the target gene TIR1. It can be synthesized to improve the germination ability of seeds under saline–alkali conditions and thus improve the salt tolerance of plants [59]. Overexpression of miR393-resistant forms of TIR1 (mTIR1) in *Arabidopsis* increases the osmoregulation and Na+ exclusion through an auxin-mediated downstream pathway, resulting in enhanced salt stress tolerance in *Arabidopsis* [60]. The *ASTIR1* gene was downregulated in annual creeping grass transgenic plants overexpressing the OS-miR393 [61]. How miR393, which is related to ground-grown *Chrysanthemum*, regulates the salt tolerance response under salt stress and how it interacts with growth hormone synthesis remains to be further verified. The cca-miR6111-5p_R-2_1ss20GT was also significantly differentially expressed in the roots and leaves of ground-grown Chrysanthemum, targets CIPK7 and CPK16, and CBL and CDPKs are calcium ion sensors. CIPKs are specific interacting proteins of CBL. When plants receive salt stress signals, Ca^2+^ receptors receive calcium ion signals to activate downstream protein phosphorylation (CDPK) or directly interact with downstream proteins (CBL-CIPK complex), thereby resisting abiotic stress [62,63]. AtCPK16 and AtDi19 (a stress-related gene family) are involved in stress resistance [64]. It is speculated that the ground-grown *Chrysanthemum* responds to salt stress by regulating the signaling pathway in the protein kinase pathway. PC-5p-838_6216 targets GRAS and AP2/ERF transcription factors and their target genes belong to ERF3 in AP2/ERF responsive ethylene factor, which has been shown to improve plant salt tolerance in *Arabidopsis* and *Wheat* [65,66]. Another target gene, GAI, is the DELLA subgroup in the GRAS family [67], which plays a negative regulatory role in the transduction of the gibberellin pathway [68], and gibberellin can effectively alleviate the damage caused by salt stress. Sweet potato and cotton GARS genes were upregulated under salt stress [69,70].

### 4.4. MiRNA-Mediated Phytohormone Signal Transduction Pathway Involved in the Salt Stress Response

**Figure 8:**
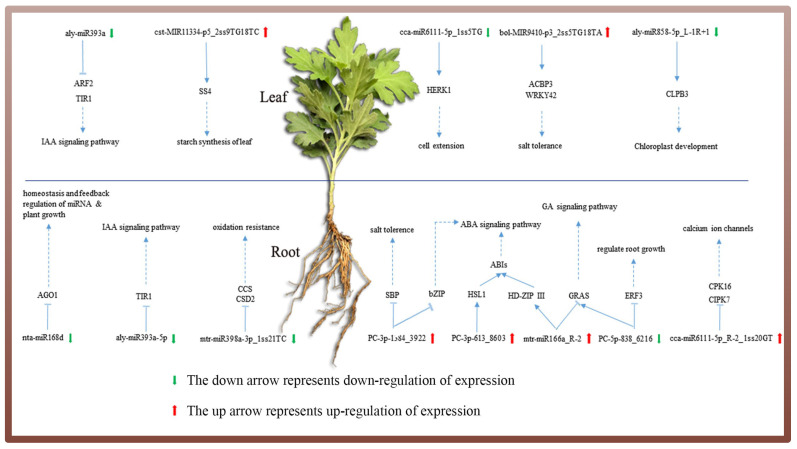
Possible responses of miRNA target genes in the pathway related to salt stress in *Chrysanthemum*.

Combined with the miRNA expression profile, degradome and transcriptome data, the target genes and their pathways were analyzed for possible effects on salt stress. Significantly differentially expressed miRNAs were found in salt-stressed roots and leaves. Most pathways in which genes are located are associated with hormone signals during plant growth and development (Figure 8).

Plant hormones are trace organic substances synthesized in plants and play important roles in plant growth and development and abiotic stress. The regulatory function in the plant hormone signaling pathway is mainly assessed in two ways: miRNA inhibits the target gene expression and the hormone response directly regulated as a key factor in the hormone signaling pathway to change the sensitivity of plants to hormone signals through the interaction between target genes and genes associated with hormone signaling pathways and participate in the regulation of plant growth and development and response to environmental signals [71,72]. In our study, growth (IAA), gibberellin (GA), abscisic acid (ABA), and brassinolide signaling (BR) in the phytohormone signaling pathway under salt stress all responded significantly. Auxin can regulate ROS levels and play a role in oxidative damage or stress signaling in plants, and Arabidopsis auxin receptor mutants have a higher tolerance to H_2_O_2_ and salt stress [73]. In the auxin signal transduction pathway, TIR1/AFB and ARF are the main auxin receptors, and miR393 affects the sensitivity of plants to auxin signals by negatively regulating the expression of the target gene TIR1/AFB [74]. Under salt stress, downregulation of aly-miR393a promotes upregulation of TIR1 and ARF genes to enhance resistance. Abscisin (ABA), a growth-inhibiting plant hormone, can induce the expression of salt stress-related genes. Such ion transporters, proteins related to the synthesis of osmotic regulators (proline, betaine synthase, etc.), and antioxidant enzymes slow down plant metabolism, thereby enhancing the tolerance of plants to salt stress [75,76]. ABA binds to PYR/PYLs to inhibit the cascade of PP2Cs kinases, by first activating SnRK2 to phosphorylate downstream sensory proteins, such as bzip transcription factors (ABFs/ABREBs, ABA-responsive elements), and then ABFs bind to the initiation of the ABA response sub-element ABRE, which in turn induces the ABA-responsive gene expression [77,78]. cca-miR6111-5p_R-2_1ss20GT targets PYR/PYLs and upregulates PYR/PYLs genes by negative regulation to promote more SnRK2 binding to ABF genes targeted by PC-3p-1384_3922 and enhancing salt tolerance in plants. In *Arabidopsis*, some components of BR signaling are directly or indirectly involved in plant responses to salt stress [79,80]. Regarding salt tolerance, enhancing BR signaling in rice can improve the salt tolerance of plants [81]. BRI1 can directly bind to brassinolide (BL) and function as a BL receptor [82] while inhibiting the BIN2 activity and promoting BR signaling [83]. The target genes of cca-miR6111-5p_1ss5TG and mtr-miR166a_R-2 in the roots of ground-grown *Chrysanthemum* coregulated the brassinolide signaling transduction pathway. The protein kinase BIN2 also acts downstream of ABA receptors and can directly phosphorylate important components of the ABA pathway SnRK2.2 and SnRK2.3, and the downstream transcription factor ABF2 [84], thereby regulating the ABA signaling pathway. Therefore, we speculate that mtr-miR166a_R-2 also regulates the ABA pathway. Moreover, different signaling pathways in plants may respond to environmental stimuli. In conclusion, under salt stress, the miRNA of ground-grown *Chrysanthemum* is involved in the regulation of phytohormone signal transduction but depends on specific miRNAs that need to be further confirmed.

## 5. Conclusions

High-throughput sequencing of the roots and leaves of *Chrysanthemum* under salt stress identified 201 known miRNAs and 79 new miRNAs belonging to 40 families. Twelve and seven miRNAs were significantly and differentially expressed in the roots and leaves. Ten miRNAs with significant differences in roots and leaves were selected and analyzed via qRT–PCR. The expression patterns of miRNAs were consistent with those detected via high-throughput sequencing. These significantly and differentially expressed miRNAs were all predicted to target 215 and 144 genes. The expression pathway analysis showed that the target genes targeted by miRNAs with significant differential expression in *Chrysanthemum* root and leaf may respond to salt stress in plants’ hormone signaling pathways, such as the auxin, abscisic acid, and rapeseed lactone signaling pathways.

## Figures and Tables

**Table 1:** Summary of small RNA sequencing data.

Treatment	Raw Reads	Clean Reads	Clean Unique Reads	Unique miRNA
SCK_L1	13,448,770	10,942,269	3,279,444 (81.65%)	192
SCK_L2	11,410,041	8,833,876	2,595,425 (81.06%)	183
SCK_L3	13,279,293	10,968,789	2,897,994 (80.56%)	194
S200_L1	16,090,479	11,374,147	2,814,510 (75.59%)	207
S200_L2	15,997,714	12,464,350	3,203,303 (79.79%)	204
S200_L3	10,850,667	7,799,571	2,202,790 (74.78%)	187
SCK_R1	10,507,148	6,672,627	2,180,643 (65.43%)	189
SCK_R2	12,656,682	8,945,834	2,852,786 (71.38%)	203
SCK_R3	13,882,772	9,815,489	2,877,824 (69.29%)	203
S200_R1	11,337,238	7,882,658	2,593,661 (68.67%)	188
S200_R2	20,096,897	14,474,914	4,047,289 (71.22%)	223
S200_R3	17,508,315	11,602,778	3,128,276 (71.31%)	210

Note: SCK_L: CK leaf; S200_L: 200 mmol/L NaCl leaf; SCK_R: CK root; S200_R: 200 mmol/L NaCl root.

**Table 2:** Significantly differentially expressed miRNAs in leaves.

	miR_name	miR_seq	Up/Down	log2	*p*-Value	Expression Level
1	aly-miR393a-5p	TCCAAAGGGATCGCATTGATCC	down	−0.81	5.70 × 10^−3^	middle
2	bol-MIR9410-p3_2ss5TG18TA	CTTTGCAGACGACTTAAATA	up	3.19	8.55 × 10^−3^	middle
3	aly-miR393a-3p	ATCATGCTATCTCTTTGGATT	down	−0.94	8.88 × 10^−3^	middle
4	cca-miR6111-5p_1ss5TG	TCTTGATGTCACGATGTATGAC	down	−3.01	2.03 × 10^−2^	middle
5	cca-MIR6111-p3_2ss17GA19CT	TTATGAAGGTAGTCTAACTCAC	up	0.62	3.90 × 10^−2^	high
6	cst-MIR11334-p5_2ss9TG18TC	TAAGGAGTGTGTAACAAC	up	2.03	4.09 × 10^−2^	middle
7	aly-miR858-5p_L-1R+1	TTCGTTGTCTGTTCGACCTTG	down	−0.53	4.56 × 10^−2^	middle

**Table 3:** Significantly differentially expressed miRNAs in roots.

	miR_Name	miR_seq	Up/ Down	log2	*p*-Value	Expression Level
1	PC-5p-838_6216	TAAACCTATCTATAACAACCT	down	−0.45	2.93 × 10^−3^	middle
2	nta-miR168d	TCGCTTGGTGCAGGTCGGGAA	up	0.31	5.02 × 10^−3^	middle
3	cca-MIR6111-p3	TTATGAAGGTAGTCTAGCCCAC	down	−0.18	5.85 × 10^−3^	middle
4	aly-miR393a-5p	TCCAAAGGGATCGCATTGATCC	down	−3.08	8.06 × 10^−3^	middle
5	PC-3p-153871_47	TGGCTCATAAGTCTCTAACTTG	up	3.33	1.11 × 10^−2^	middle
6	mtr-miR398a-5p_2ss12GC21AT	GGAGTGACACTCAGAACACATG	down	−2.86	1.45 × 10^−2^	middle
7	cca-miR6111-5p_R-2_1ss20GT	TCTTTATGTCACGATGTATT	up	1.04	2.17 × 10^−2^	middle
8	PC-3p-613_8603	TTTAAGTAGTGGACAATTGGA	up	0.7	2.32 × 10^−2^	middle
9	mtr-miR166a_R-2	TCGGACCAGGCTTCATTCC	up	0.27	2.65 × 10^−2^	middle
10	PC-3p-1384_3922	TCCACTCTGCTTTCTCTGAGGT	up	0.41	3.60 × 10^−2^	middle
11	mtr-miR398a-3p_1ss21TC	TGTGTTCTCAGGTCACCCCTC	down	−1.14	4.50 × 10^−2^	middle
12	ath-miR162a-5p_L-1_2ss5GT6GA	GGATACAGCGGTTCATCGATC	down	−0.58	4.97 × 10^−2^	middle

**Table 4:** Number of genes and pathway names with the largest distribution of target genes in the KEGG pathway.

Tissue	KEGG ID	KEGG Name	Gene Number
	ko04075	Plant hormone signal transduction	11
	ko04626	Plant–pathogen interaction	4
S200-L vs. CK-L	ko00260	Glycine, serine, and threonine metabolism	4
	ko04120	Ubiquitin-mediated proteolysis	4
	ko04075	Plant hormone signal transduction	11
	ko03013	RNA transport	6
S200-R vs. CK-R	ko03015	mRNA surveillance pathway	4
	ko04144	Endocytosis	4

## Data Availability

All data generated or analyzed during this study are included in this published article and its Supplementary Materials.

## Supplementary Materials

The following supporting information can be downloaded at: https://www.mdpi.com/article/10.3390/genes14030561/s1, Table S1: Distribution of conserved miRNAs in *Chrysanthemum*; Table S2: Novel miRNAs identified in *Chrysanthemum*; Table S3: Degradome sequencing results of raw reads and unique mapped reads in four libraries; Table S4: Analysis of transcripts targeted by miRNAs; Table S5: Categories of transcripts in the roots and leaves of *Chrysanthemum*; Table S6: GO functional annotation of target genes in *Chrysanthemum*; Table S7: Functional analysis of target genes of miRNAs differentially expressed in roots and leaves of *Chrysanthemum* under salt stress; Figure S1: Length distribution of sequencing (a) total miRNAs and (b) unique miRNAs; Figure S2: Prediction of new miRNA stem-loop structures in *Chrysanthemum*; Figure S3: Specific analysis of nucleotide positions; Figure S4: The most significant functions among the top 20 GO terms in (a) roots and (b) leaves.
