# Supplementary material for: Identification of Small RNAs Associated with Salt Stress in Chrysanthemums through High-Throughput Sequencing and Bioinformatics Analysis
Source: Genes (Basel). 2023 Feb 23;14(3):561. doi: 10.3390/genes14030561 (PMC10048073; doi:10.3390/genes14030561)
Supplement: Supplementary file 1 [file genes-14-00561-s001.zip › Description of supplementary materials.pdf]

Supplementary materials

Table S1 Distribution of conserved miRNAs in *Chrysanthemum*

Table S2 Novel miRNAs identified in *Chrysanthemum*

Table S3 Degradome sequencing results of raw reads and unique mapped reads in four libraries

Table S4 Analysis of transcripts targeted by miRNAs

Table S5 Categories of transcripts in the roots and leaves of *Chrysanthemum*

Table S6 GO functional annotation of target genes in *Chrysanthemum*

Table S7 Functional analysis of target genes of miRNAs differentially expressed in roots and leaves of *Chrysanthemum* under salt stress

Figure S1 Length distribution of sequencing (a) total miRNAs and (b) unique miRNAs

Figure S2 (1)and Figure S2 (2) Prediction of new miRNA stem-loop structures in *Chrysanthemum*

Figure S3 Specific analysis of nucleotide positions

Figure S4 The most significant functions among the top 20 GO terms in (a) roots and (b) leaves
