# Supplementary material for: Identification of Small RNAs Associated with Salt Stress in Chrysanthemums through High-Throughput Sequencing and Bioinformatics Analysis
Source: Genes (Basel). 2023 Feb 23;14(3):561. doi: 10.3390/genes14030561 (PMC10048073; doi:10.3390/genes14030561)
Supplement: Supplementary file 1 [file genes-14-00561-s001.zip › figure S1.pdf]

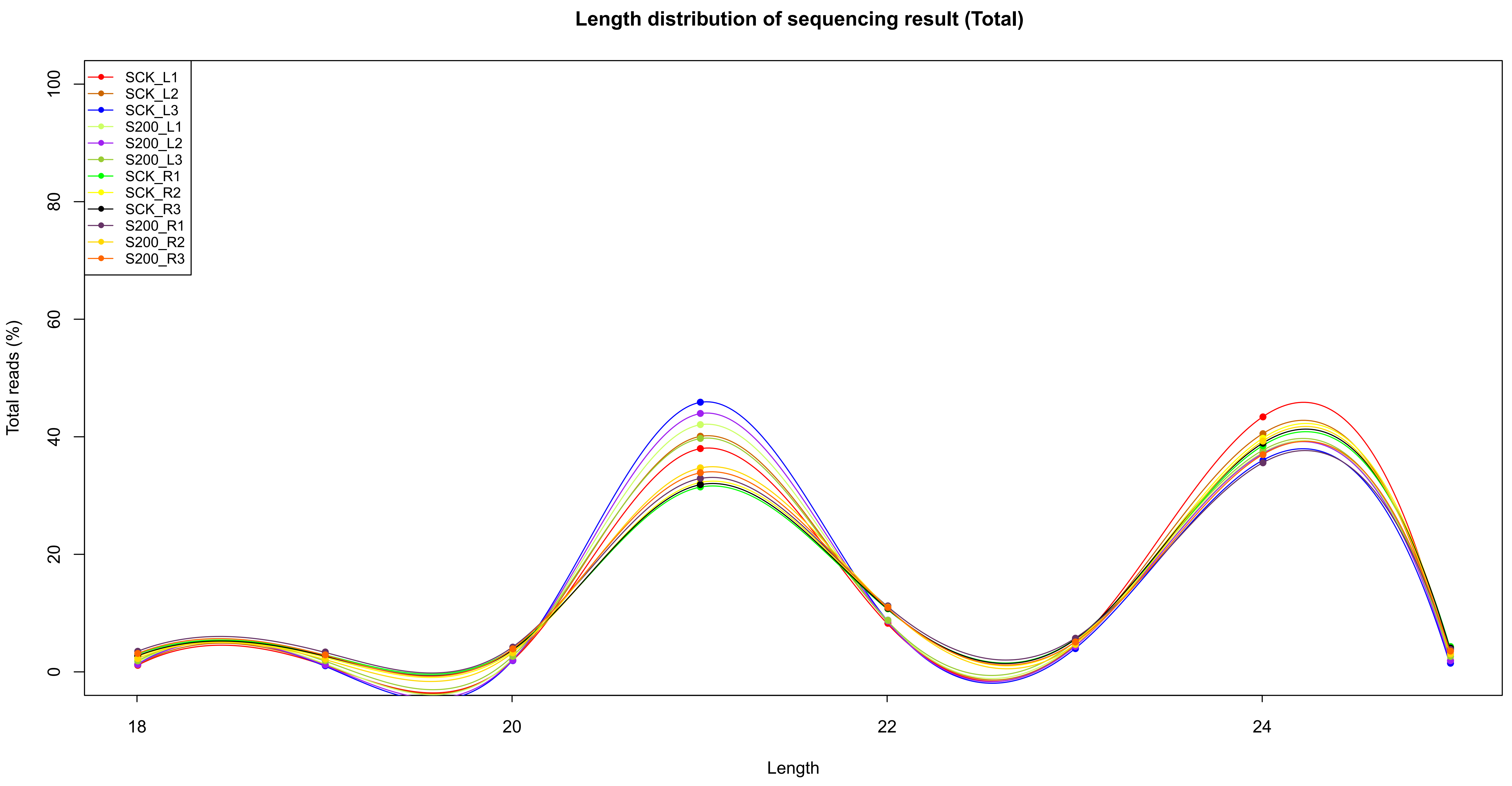
Length distribution of sequencing result (Total)
Total reads (%)
Length
0
20
40
60
80
100
18
20
22
24
SCK_L1
SCK_L2
SCK_L3
S200_L1
S200_L2
S200_L3
SCK_R1
SCK_R2
SCK_R3
S200_R1
S200_R2
S200_R3

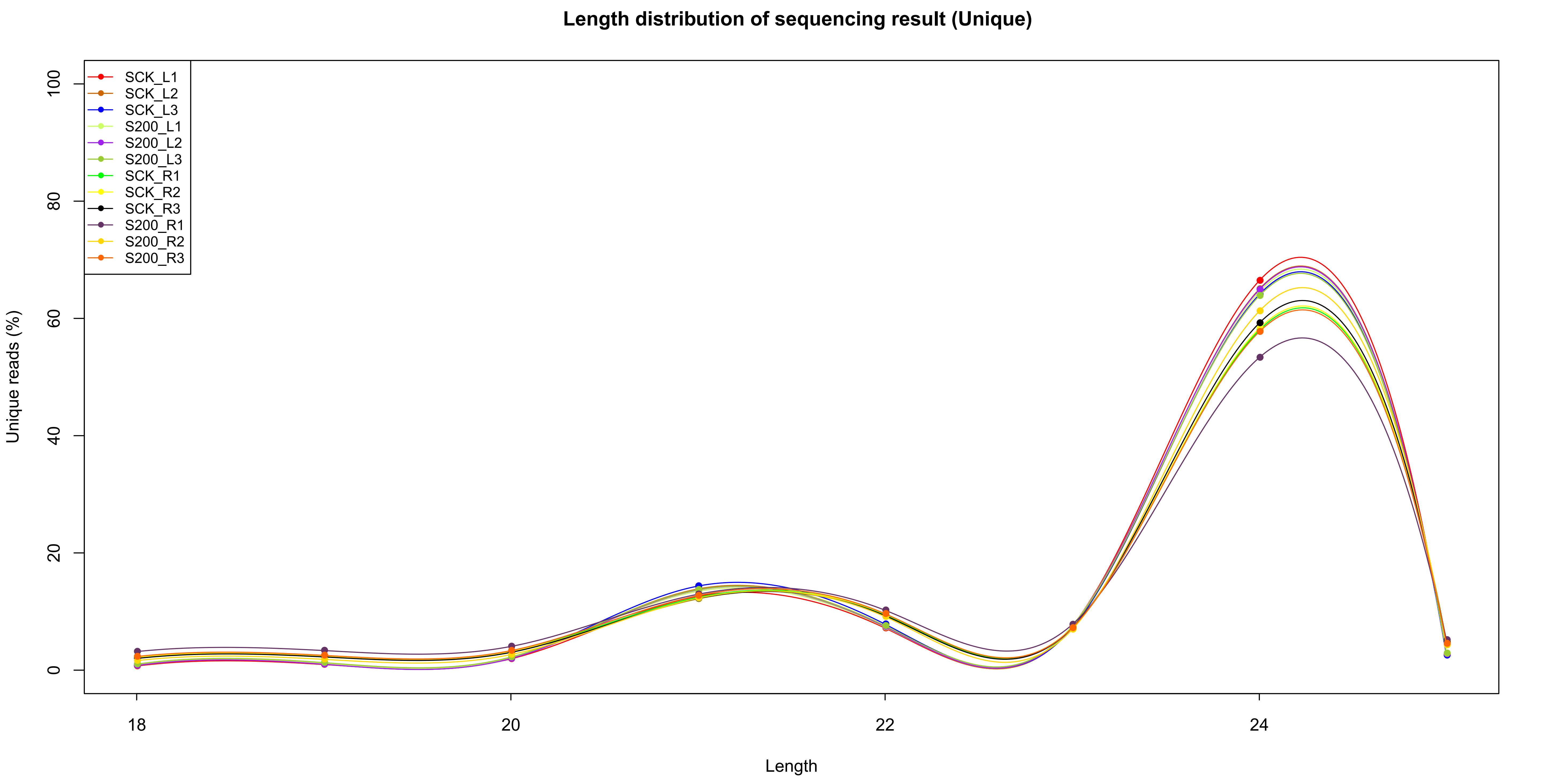
Length distribution of sequencing result (Unique)
SCK_L1
SCK_L2
SCK_L3
S200_L1
S200_L2
S200_L3
SCK_R1
SCK_R2
SCK_R3
S200_R1
S200_R2
S200_R3
Unique reads (%)
0
20
40
60
80
100
18
20
22
24
Length
