# Supplementary material for: Identification of Small RNAs Associated with Salt Stress in Chrysanthemums through High-Throughput Sequencing and Bioinformatics Analysis
Source: Genes (Basel). 2023 Feb 23;14(3):561. doi: 10.3390/genes14030561 (PMC10048073; doi:10.3390/genes14030561)
Supplement: Supplementary file 1 [file genes-14-00561-s001.zip › figure S2 (2).pdf]

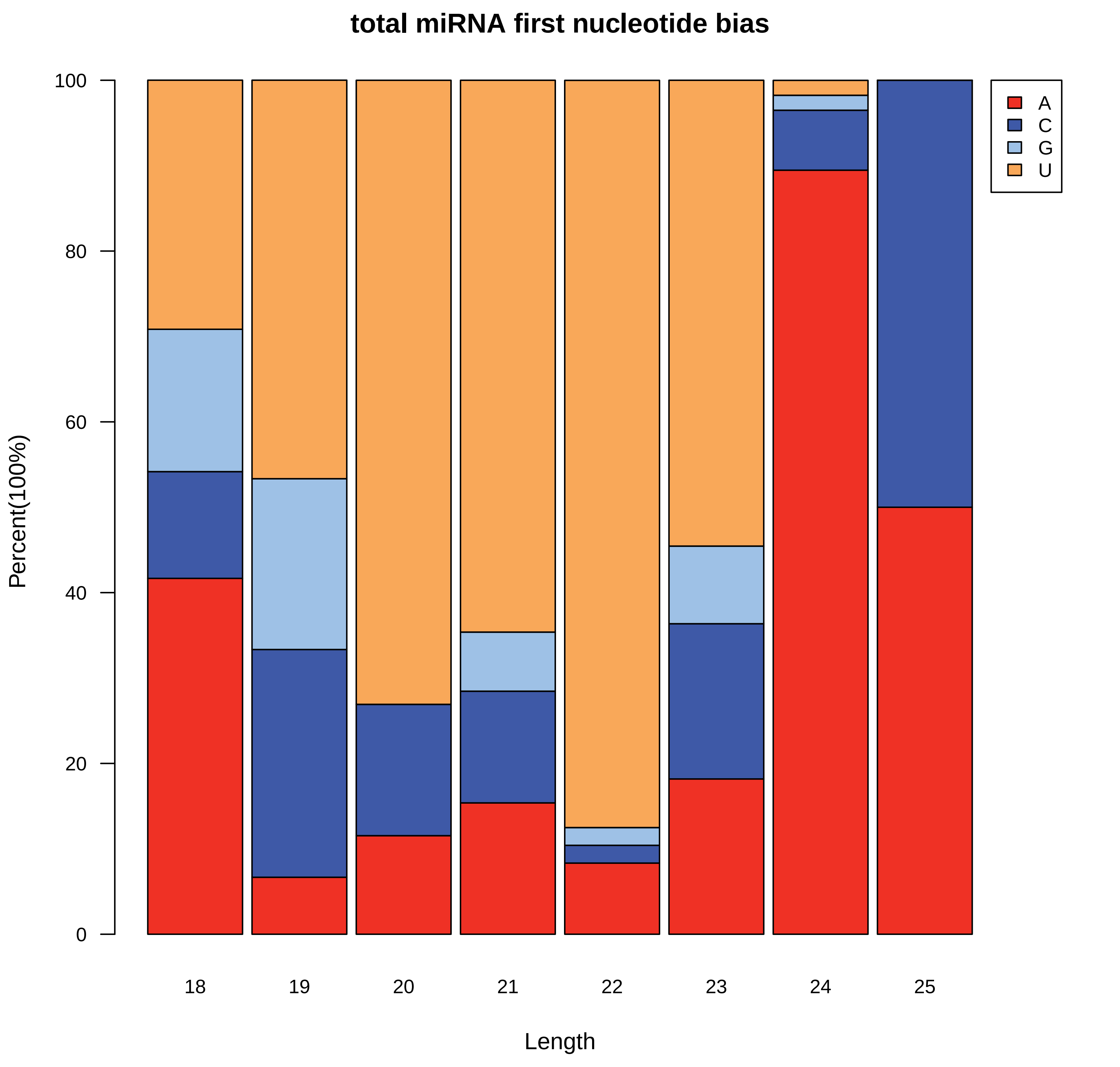
total miRNA first nucleotide bias
Percent(100%)
0
20
40
60
80
100
18
19
20
21
22
23
24
25
Length
A
C
G
U

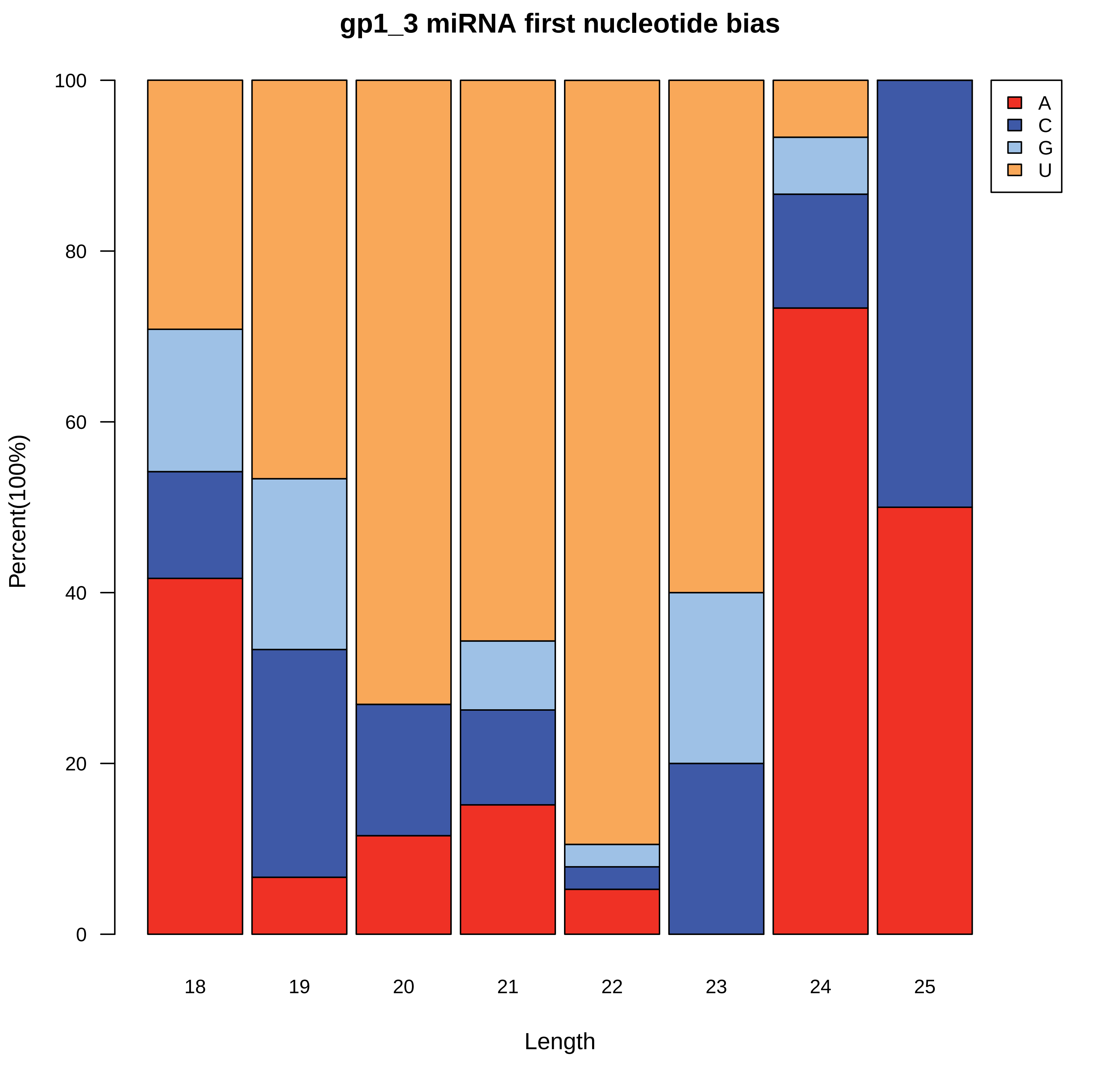
gp1_3 miRNA first nucleotide bias
Percent(100%)
100
80
60
40
20
0
18
19
20
21
22
23
24
25
Length
A
C
G
U

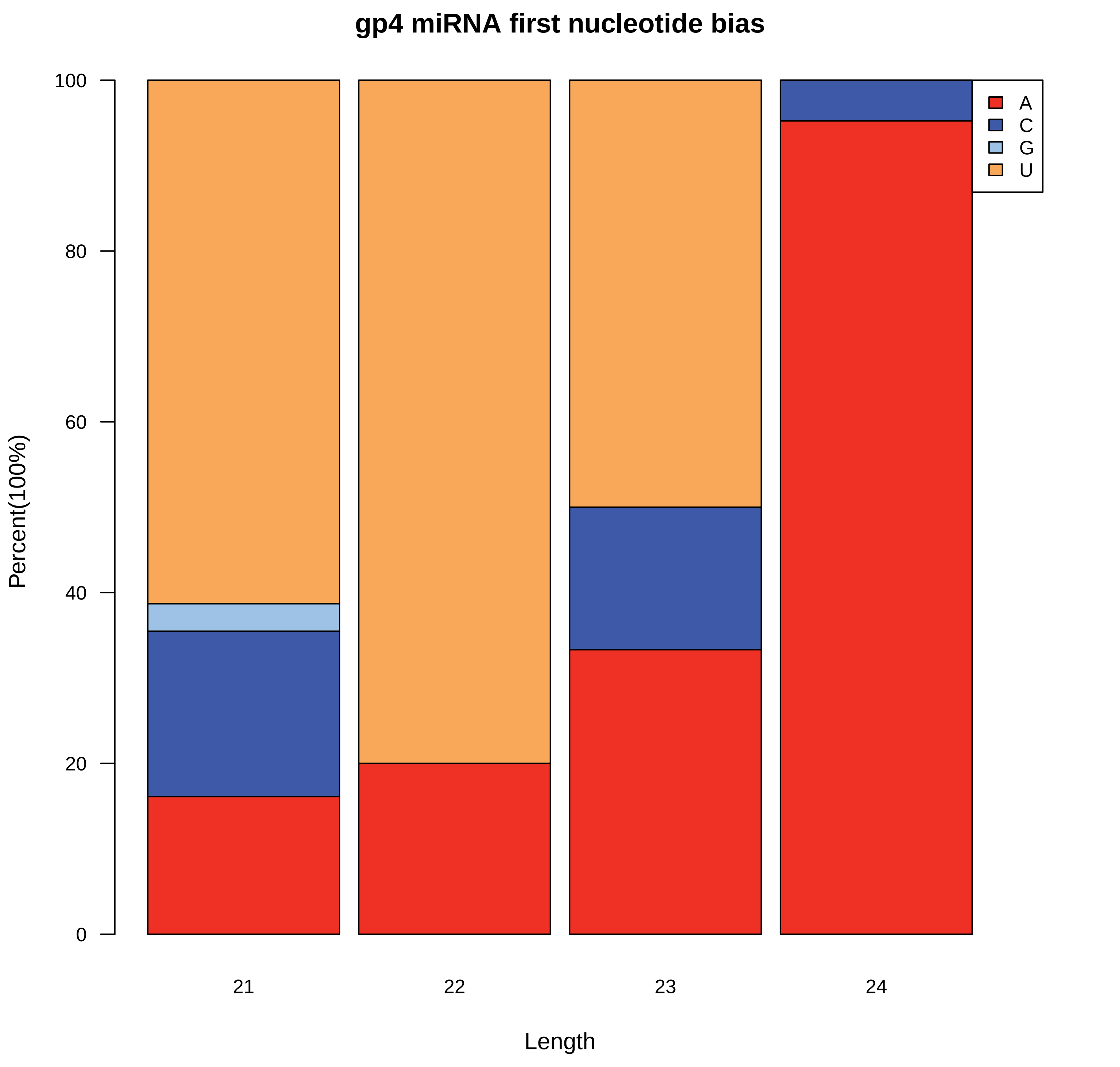
gp4 miRNA first nucleotide bias
Percent(100%)
0
20
40
60
80
100
21
22
23
24
Length
A
C
G
U
