# Supplementary figures and images for: Identification of Small RNAs Associated with Salt Stress in Chrysanthemums through High-Throughput Sequencing and Bioinformatics Analysis
Source: Genes (Basel). 2023 Feb 23;14(3):561. doi: 10.3390/genes14030561 (PMC10048073; doi:10.3390/genes14030561)

# total miRNA nucleotide bias

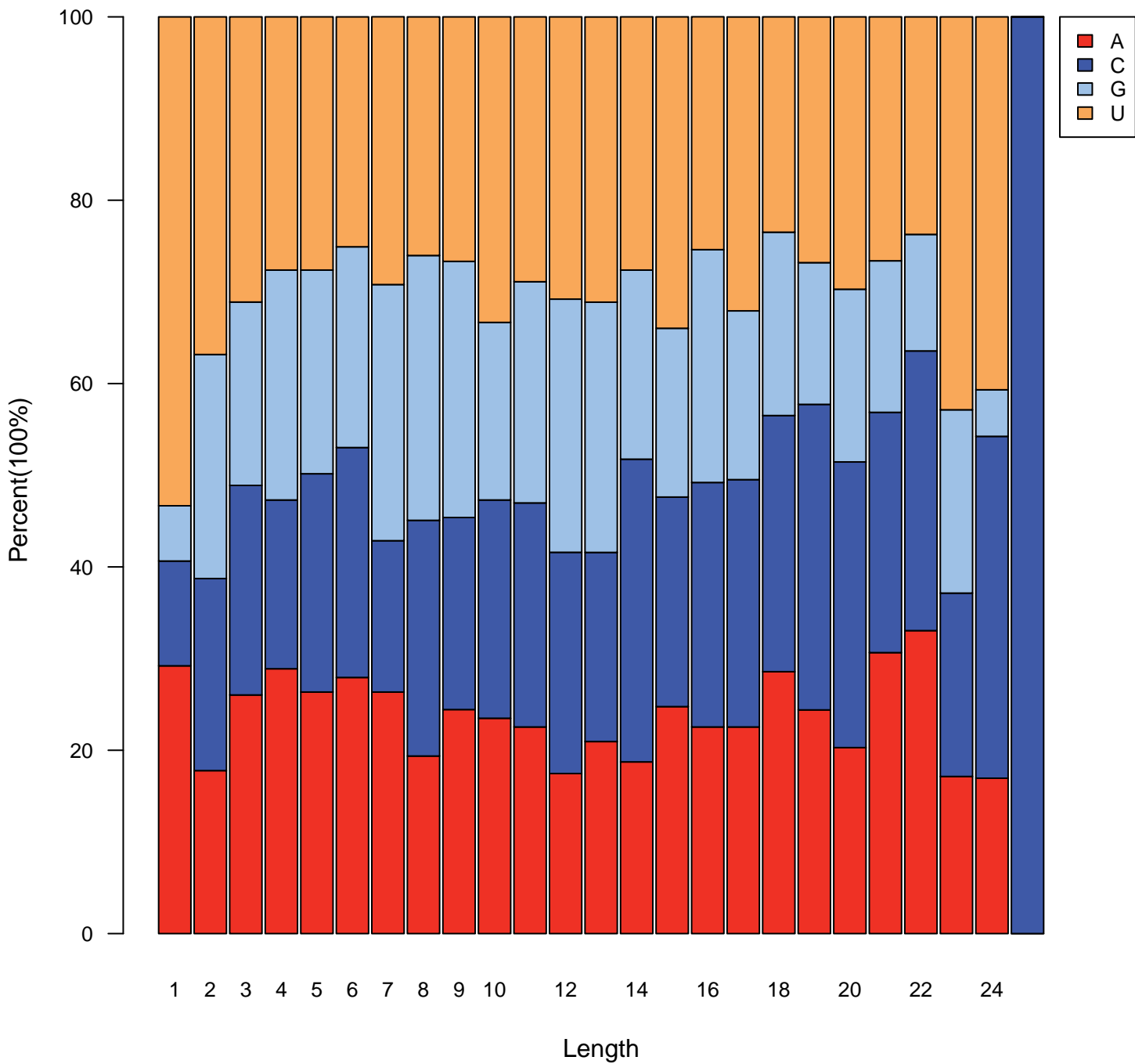

# gp1\_3 miRNA nucleotide bias

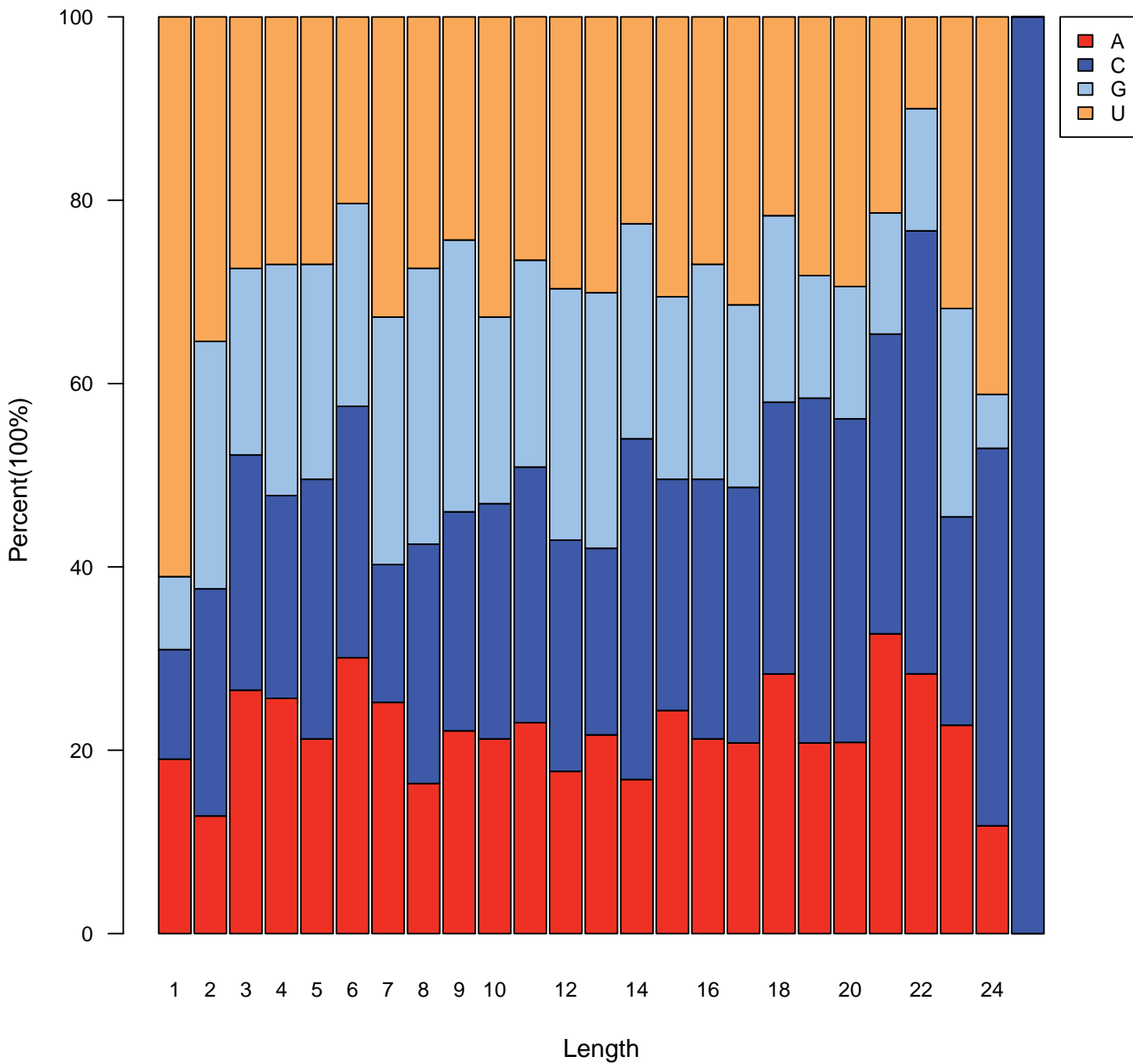

# gp4 miRNA nucleotide bias

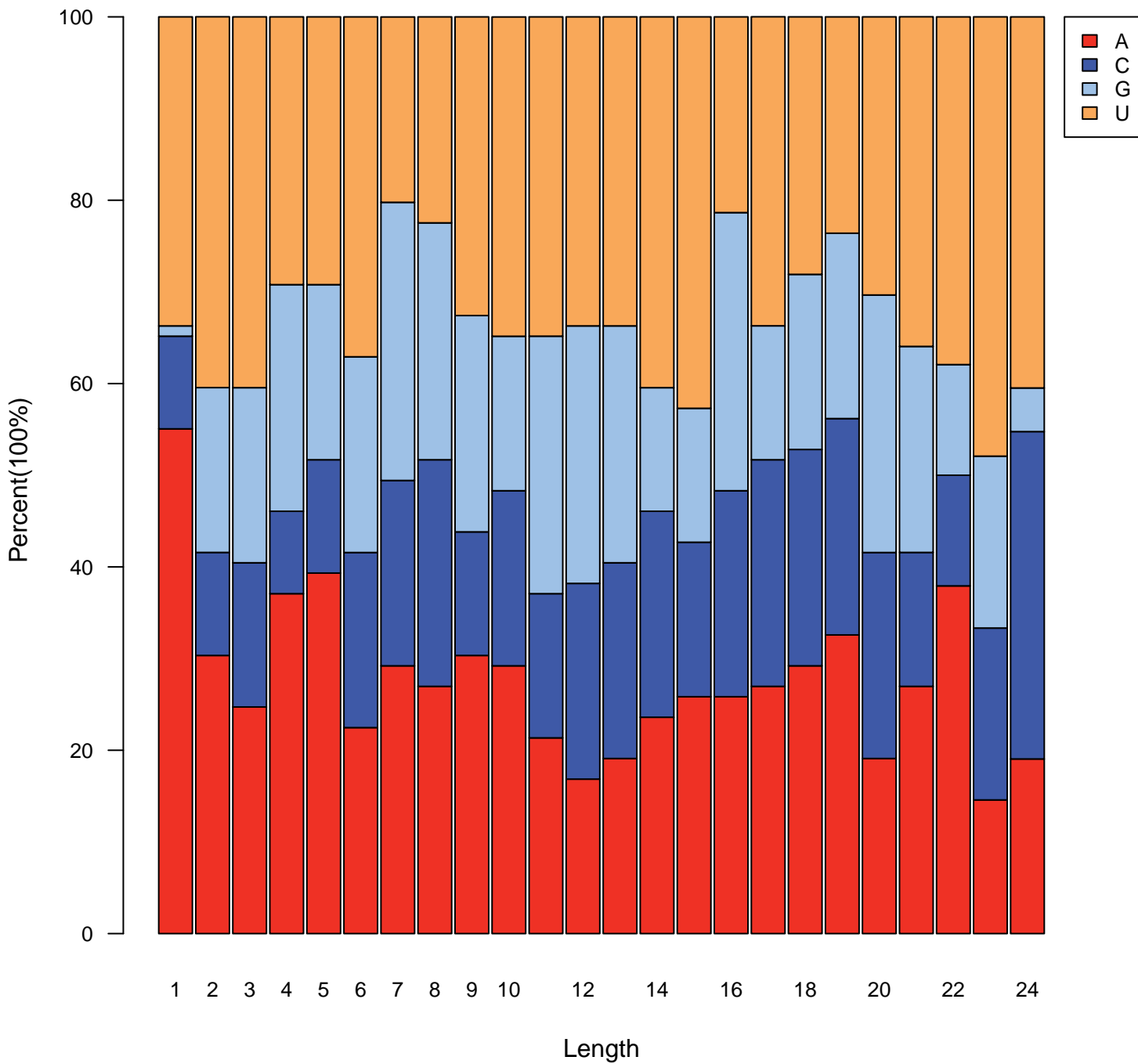

Supplement: Supplementary file 1 [file genes-14-00561-s001.zip › figure S2 (1).pdf]

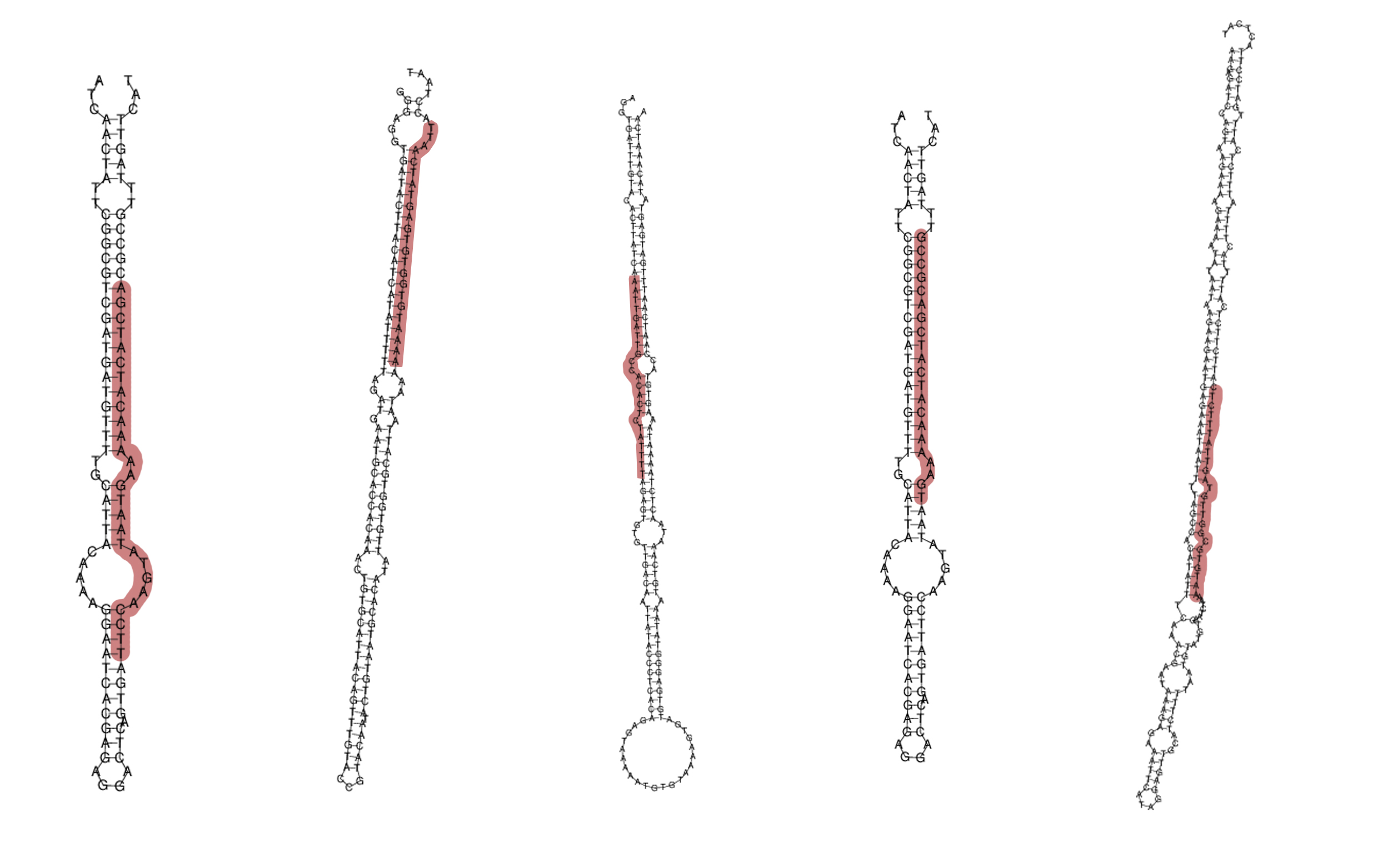

Supplement: Supplementary file 1 [file genes-14-00561-s001.zip › figure S3.jpg]

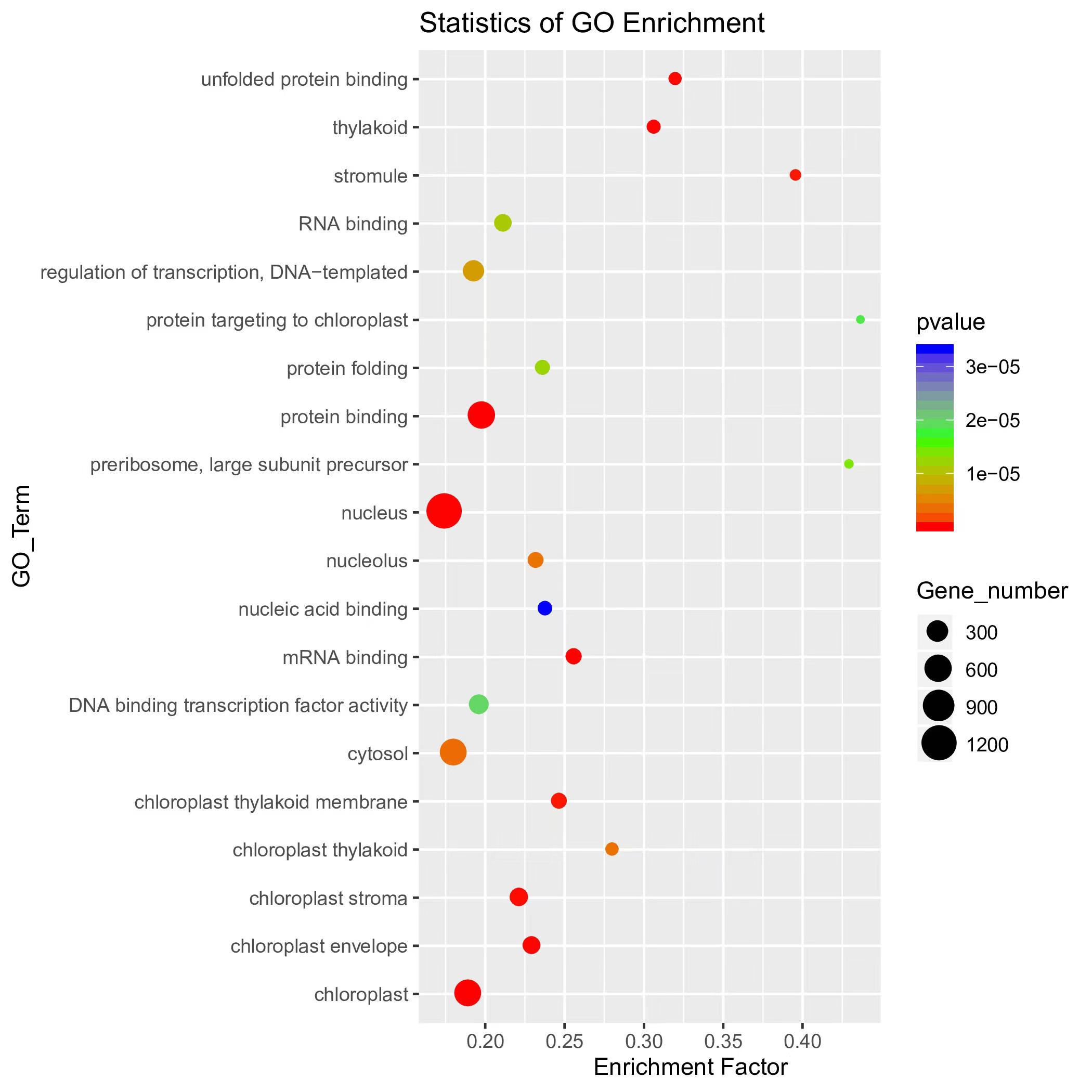

Supplement: Supplementary file 1 [file genes-14-00561-s001.zip › figure S4a.jpg]

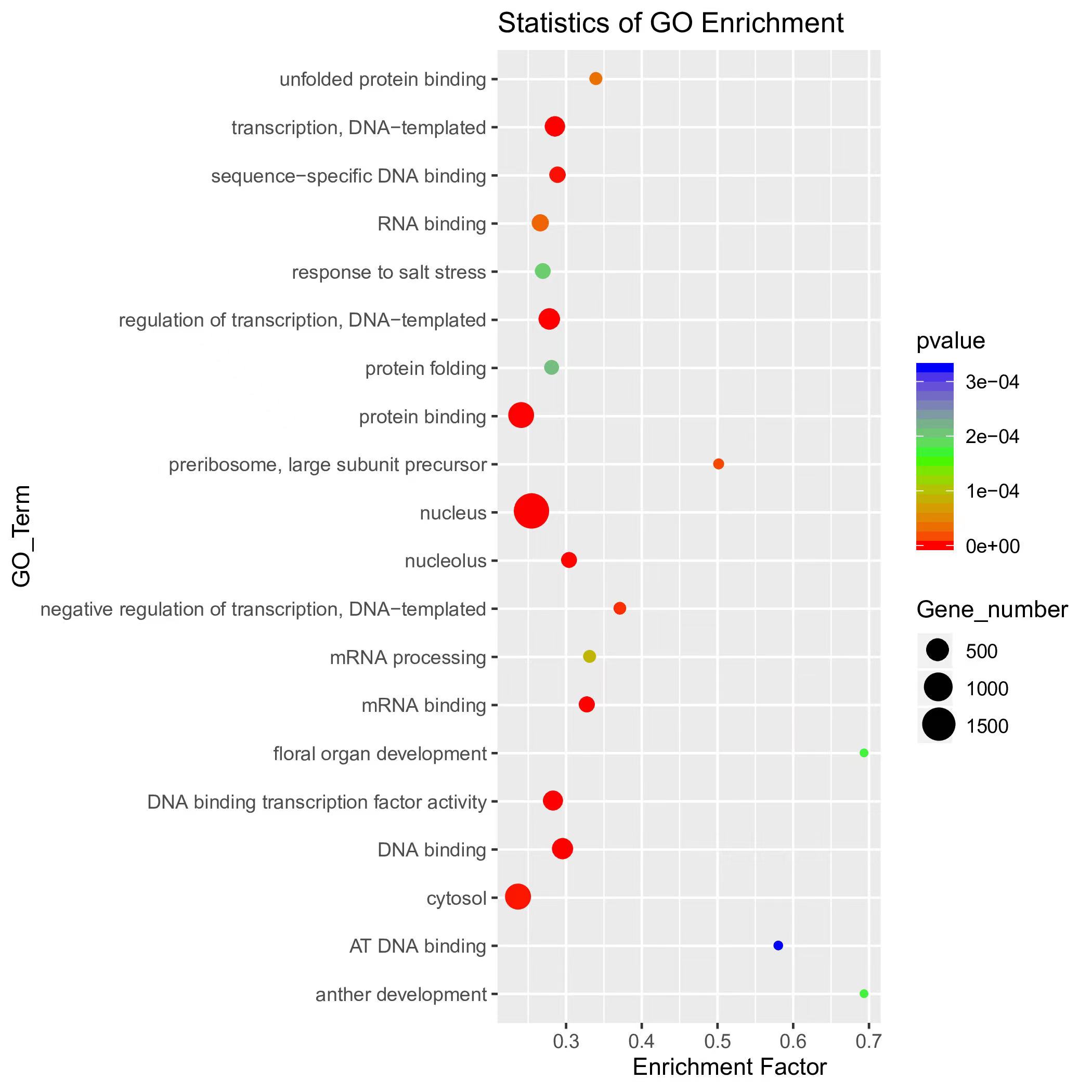

Supplement: Supplementary file 1 [file genes-14-00561-s001.zip › figure S4b.jpg]
